# Telocytes and putative stem cells in ageing human heart

**DOI:** 10.1111/jcmm.12509

**Published:** 2014-12-25

**Authors:** Laurentiu M Popescu, Antoanela Curici, Enshi Wang, Hao Zhang, Shengshou Hu, Mihaela Gherghiceanu

**Affiliations:** a Department of Cellular and Molecular Medicine, ‘Carol Davila’ University of Medicine and PharmacyBucharest, Romania; b Division of Advanced Studies, ‘Victor Babeş’ National Institute of PathologyBucharest, Romania; c State Key Laboratory of Cardiovascular Disease, Center for Pediatric Cardiac Surgery, Fuwai Hospital and National Center for Cardiovascular Diseases, Chinese Academy of Medical SciencesBeijing, China; d Laboratory for Electron Microscopy, ‘Victor Babeş’ National Institute of PathologyBucharest, Romania

**Keywords:** telocytes, cardiac stem cells, cardiomyocytes, fibroblasts, macrophages, lipofuscin granules, Schwann cells, human ageing heart

## Abstract

Tradition considers that mammalian heart consists of about 70% non-myocytes (interstitial cells) and 30% cardiomyocytes (CMs). Anyway, the presence of telocytes (TCs) has been overlooked, since they were described in 2010 (visit http://www.telocytes.com). Also, the number of cardiac stem cells (CSCs) has not accurately estimated in humans during ageing. We used electron microscopy to identify and estimate the number of cells in human atrial myocardium (appendages). Three age-related groups were studied: *newborns* (17 days–1 year), *children* (6–17 years) and *adults* (34–60 years). Morphometry was performed on low-magnification electron microscope images using computer-assisted technology. We found that interstitial area gradually increases with age from 31.3 ± 4.9% in newborns to 41 ± 5.2% in adults. Also, the number of blood capillaries (per mm^2^) increased with several hundreds in children and adults *versus* newborns. CMs are the most numerous cells, representing 76% in newborns, 88% in children and 86% in adults. Images of CMs mitoses were seen in the 17-day newborns. Interestingly, no lipofuscin granules were found in CMs of human newborns and children. The percentage of cells that occupy interstitium were (depending on age): endothelial cells 52–62%; vascular smooth muscle cells and pericytes 22–28%, Schwann cells with nerve endings 6–7%, fibroblasts 3–10%, macrophages 1–8%, TCs about 1% and stem cells less than 1%. We cannot confirm the popular belief that cardiac fibroblasts are the most prevalent cell type in the heart and account for about 20% of myocardial volume. Numerically, TCs represent a small fraction of human cardiac interstitial cells, but because of their extensive telopodes, they achieve a 3D network that, for instance, supports CSCs. The myocardial (very) low capability to regenerate may be explained by the number of CSCs, which decreases fivefold by age (from 0.5% to 0.1% in newborns *versus* adults).

## Introduction

Abundant data exist concerning the estimation of cell number in mammal hearts (a Google Scholar search returns about 14,000 results!). However, the dogma that interstitial cells are more numerous than cardiomyocytes (CMs) is quite popular. The tradition considers that, numerically, the human myocardial pump consists of about 70% non-myocytes (interstitial cells) and 30% CMs [1,2].

Studies by Zak and Nag [3–5] during the 1970s defined and quantified the cellular populations of the adult rat left ventricle. These were based on morphological features using electron microscopy (EM) and gradient centrifugation. The results obtained on specific cardiac regions were further extrapolated to the entire heart and now, it is assumed that CMs represent only 30–40% from total cells in normal heart [6–9]. Five types of non-muscle cells were described using the dissociation of adult heart into single-cell suspensions: endothelial cells, fibroblasts, pericytes, smooth muscle cells and macrophages [4,5].

Now telocytes (TCs) are accepted as a distinct type of interstitial cells (visit http://www.telocytes.com; Wikipedia) in heart [10–29]. FIB-SEM tomography, the most promising approach for 3D imaging at the subcellular level [30] confirmed the existence of cardiac TCs [31]. TCs are featured by their extremely long cellular prolongations (tens to hundreds of μm) termed telopodes (Tps). These Tps have an alternation of very thin segments (below the resolving power of light microscopy) named podomers and dilated portions named podoms. TCs were found in myocardium as a 3D network among the bundles of CMs, blood capillaries, nerve endings and immunoreactive cells [12,18,19,21,31–33]. TCs are key-players in inter(trans)-cellular signalling [18,21]. Beyond the specific ultrastructural ‘portrait’ and immunophenotype or electrophysiological properties [22,34,35], the individuality of TCs (*e.g*. in comparison with fibroblasts) was shown by the microRNA imprint [36] gene profile [37–39] or proteomics [40]. The so-called interstitial Cajal-like cells described previously (2005–2009) in heart [41–47] correspond more or less to TCs, but nevertheless TCs are not Cajal-like cells. Their characteristics are different from the classical cells of Cajal of the gastro-intestinal tract. In 2010, became obviously that TCs represent a distinct (novel) type of interstitial cells. The shortest definition of TCs is: cells with Tps [21]. A very recent review ‘Telocyte revisited’ is available [48].

Telocytes seem to provide support for cardiac stem cells (CSCs) in their niches [11,18]. TCs presumably guide tissue integration of myocardial precursors [32,46] and participate in neoangiogenesis [49,50]. Recently, Gherghiceanu and Popescu [18,51] described heterocellular junctions formed by TCs with CSCs or CMs or other interstitial cells in the rat and human adult heart. Such junctions ‘do not fit the scheme’ [52].

This study investigated the number of interstitial cells in ageing human heart with emphasis on TCs and CSCs. The results suggest that TCs could form an integrative interstitial cardiac system, which may be essential for the decision of CSCs to proliferate, differentiate and maturate into new CMs. However, this capability decreases by ageing.

## Materials and methods

### Patients

Fourteen patients aged between 17 days and 60 years suffering from heart diseases were recruited from Fuwai Hospital, Beijing. All studied patients who underwent cardiac surgery were of Chinese Han nationality. Patients with syndromic congenital heart diseases (CHD) such as Marfan, Noonan, Holt-Oram, Alagille and CHARGE syndromes, as well as chromosomal abnormalities that are highly associated with CHD (*e.g*. 21-trisomy or 22q11.2 deletion) were excluded from this study. Patients were divided into three age groups: newborns (five cases); children (four cases); and adults (five cases) (Table 1). The study was performed according to the World Medical Association Declaration of Helsinki and the terms required by the Research Ethics Committee of The Chinese Academy of Medical Sciences; written informed consent was obtained from all participants or relatives.

**Table 1:** Clinical data of patients

Group	Sex	Age	Clinical diagnosis
Newborns	M	17 days	CHD, TGA, ASD
	F	6 months	CHD, VSD,ASD,PFO
	M	8 months	CHD, ASD,TI
	M	8 months	CHD, VSD, PFO, MI
	M	8 months	CHD, VSD, DCRV
Children	F	6 years	CHD, ASD
	F	12 years	CHD, TOF
	F	15 years	CHD, VSD
	M	17 years	CHD, PS, TI, PH
Adults	F	34 years	CHD, PS
	M	45 years	CHD, ASD
	M	55 years	MS
	M	57 years	MS
	M	60 years	MS

CHD: congenital heart disease; TGA: Transposition of the great arteries; ASD: Atrial septal defect; VSD: Ventricular septal defect; PFO: Patent foramen ovale; TI: Tricuspid insufficiency; MI: Mitral insufficiency; DCRV: Double chamber of right ventricle; TOF: Tetralogy of Fallot; PS: Pulmonary stenosis; PH: Pulmonary hypertension; MS: Mitral stenosis.

### Electron microscopy

Right atrial appendage fragments used in this study were obtained from CHD patients before cardiopulmonary bypass. Tissue samples were cut into about 1-mm^3^ fragments and fixed by immersion for 4 hrs in 4% glutaraldehyde in 0.1 M cacodylate buffer, pH 7.4 at 4°C. After two baths in 0.1 M cacodylate buffer, the samples were post-fixed for 1 hr in 1% OsO_4_ with 1.5% K_4_Fe(CN)_6_ (potassium ferrocyanide-reduced osmium) in 0.1 M cacodylate buffer, at room temperature. Afterwards, the samples were dehydrated through increasing ethanol and embedded in Epon 812 at 60°C for 48 hrs [44,53]. Semi-thin sections (1-μm thick) were stained with toluidine blue and examined under light microscope Nikon Eclipse E600 (Nikon Instruments, Inc., Tokyo, Japan) equipped with charge-coupled device (CCD) AxioCamHRc (Zeiss). Thin sections (50–60 nm) were examined with a Morgagni 286 transmission electron microscope (FEI Company, Eindhoven, The Netherlands) at 80 kV. Digital electron micrographs were recorded with Mega View III CCD using iTEM SIS software (Olympus, Soft Imaging System, Münster, Germany).

### Morphometric analysis

The morphometry was performed on EM images with iTEM SIS software (Olympus, Soft Imaging System) using 300 randomly selected images.

We took into account the state-of-the-art morphometry for better quantitative 3D morphology in cardiac muscle [54].

iTEM SIS software was used to count and measure cells on EM images with low magnifications: 4400×, 7100× and 8900×. Higher magnifications (11,000×, 18,000× and 22,000×) were used to determine subcellular components. Light microscope images (sections stained with toluidine blue) were analysed using ImageJ to determine the interstitial area and diameter of CMs.

All data were statistically analysed and Student's *t*-test was used to define differences between groups. *P* < 0.05 was considered significant. Data are presented as the mean with SD.

## Results

**Fig. 1:**
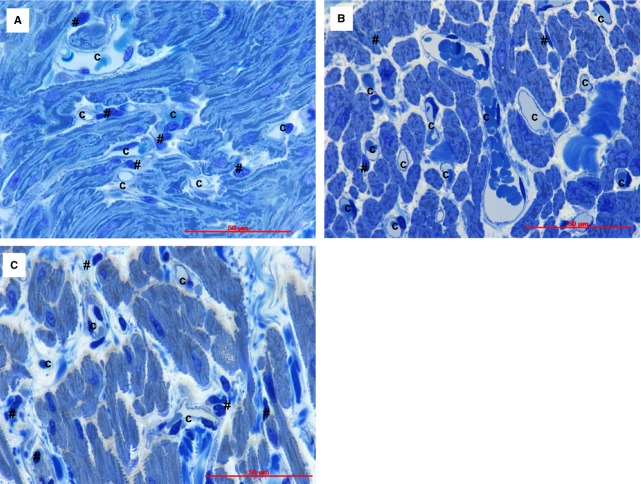
Human atrial tissue from newborns (**A**); children (**B**) and adults (**C**). Light microscopy images of semi-thin sections stained with toluidine blue show capillaries (C) which are easily visible in the cardiac interstitium. The interstitial cells (#) cannot be discriminated under light microscope. Bars 50 μm.

**Fig. 2:**
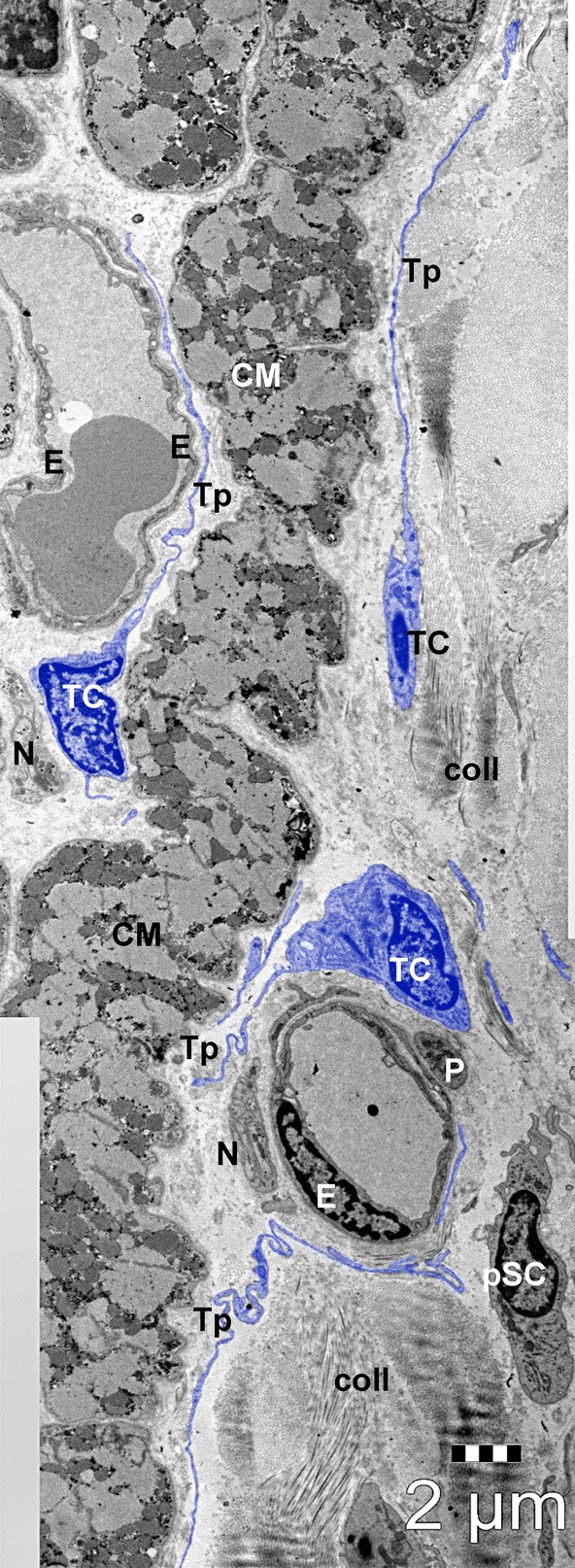
General view of human atrial interstitium (8-months-old patient) where telocytes (TC) with telopodes (Tp), endothelial cells (E), pericytes (P), nerve endings (N) and putative stem cells (pSC) could be seen on electron microscopy. Cardiomyocytes (CM); coll – collagen. Bar 2 μm.

Light microscopy of myocardium (Fig. 1) shows that the interstitial area increases progressively with age from 31.3 ± 4.9% in newborns to 33.8 ± 5.6 in children and 41 ± 5.2 in adults (*P* < 0.05 and *P* < 0.01, respectively). As percentage, this would represent a gradual increase in non-myocyte space: about 2% for children and 10% for adults. Also, the number of blood capillaries per mm^2^ (Table 2) increased several hundreds in children and adults *versus* newborns (*P* < 0.001). However, the diameter of capillary lumen was not (at all) changed. Figure 1 shows that the light microscopy (even using of 1-μm sections) does not allow the precise identification of interstitial cell type because of the limited resolving power (0.2 μm). Thus, we used transmission electron microscopy to identify and quantify the interstitial non-CM cells (Table 3, Fig. 2).

**Table 2:** Blood capillary morphometry in human atrial myocardium (mean ± SE)

Capillaries	Newborns	Children	Adults
Diameter (μm)	6.2 ± 1.9	6.2 ± 1.6	6.5 ± 1.5
Density (no/mm)	910 ± 105	1217 ± 171*	1306 ± 137*,†

* The number significantly *increased* in children and adults compared with newborns (*P* < 0.001).

† The difference between capillary density in children and adults is not significant (*P* > 0.05).

**Table 3:** Number of cardiomyocytes and interstitial cells in human ageing heart (atria) (mean ± SD)

Number/mm^2^	Newborns	Children	Adults
Cardiomyocytes	11,223 ± 2460	28,567 ± 5920*	24,817 ± 3260*,†
Endothelial cells	1930 ± 140	2340 ± 280*	2170 ± 210*
Vascular smooth muscle cells & pericytes	1014 ± 11	958 ± 10‡	896 ± 12†,‡
Schwann cells	261 ± 78	271 ± 89	280 ± 73*
Fibroblasts	241 ± 50	112 ± 11‡	438 ± 56*,§
Macrophages	125 ± 12	53 ± 7‡	350 ± 17*,§
Telocytes	22 ± 2	27 ± 4‡	19 ± 3†,‡
Putative stem cells	17 ± 3	7 ± 1‡	4 ± 1†,‡

* The number of cells significantly *increased* in children and adults compared with newborns (*P* < 0.01).

† The number of cells significantly *decreased* in adults compared with children (*P* < 0.02).

‡ The number of cells significantly *decreased* in children and adults compared with newborn group (*P* < 0.01).

§ The number of cells significantly *increased* in adults compared with children (*P* < 0.001).

**Fig. 3:**
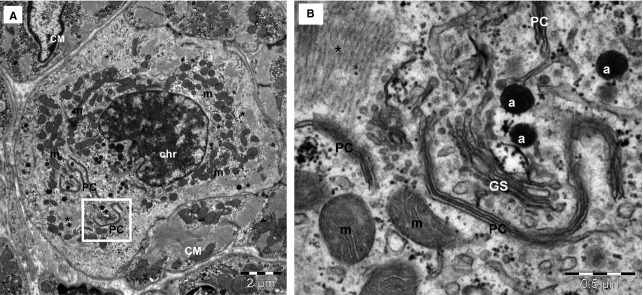
TEM image of atrial tissue (17-days-old patient) shows a cardiomyocyte undergoing mitosis (**A**). The nuclear chromatin (chr) is coarsely clumped into the nucleus and ‘*paired cisternae*’ (PC) of the nuclear envelope with endoplasmic reticulum are visible into the cytoplasm. Mitotic cardiomyocyte has high number of mitochondria (m) and very few myofilaments (*). (**B**) Higher magnification of square-marked area in (**A**) shows characteristic feature for prophase: the nuclear envelope fragments in parallel pairs with ER cisternae in the cytoplasm (PC). a – atrial granules; GS– Golgi system. Bars 2 μm (**A**), 0.5 μm (**B**).

**Fig. 4:**
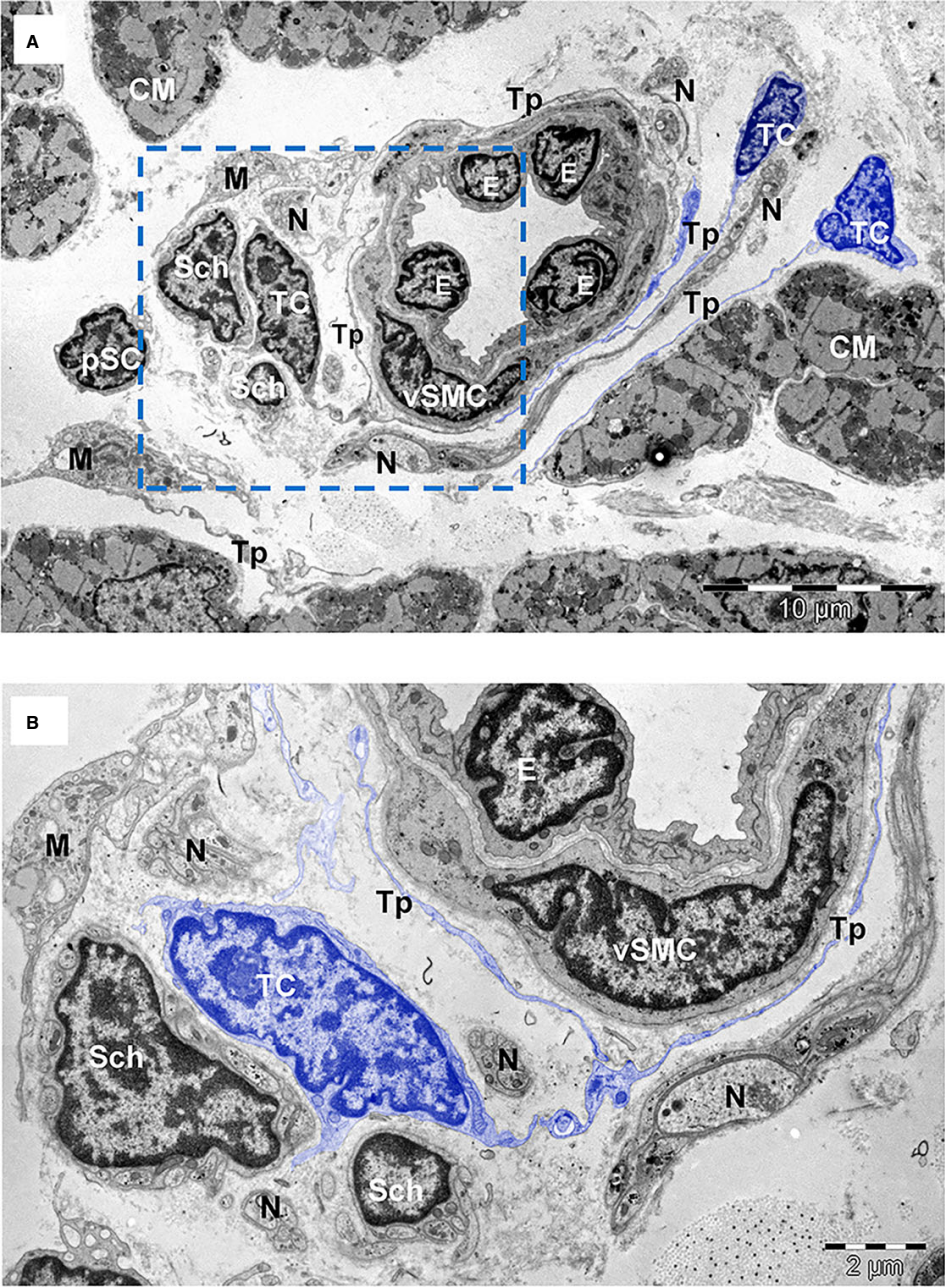
(**A**) TEM image of human atrial interstitium (8-months-old patient) shows telocytes (TC) with long and thin processes (Tp) running around a small artery with endothelial cells (E) and vascular smooth muscle cells (vSMC). There are also visible Schwann cells (Sch), nerve endings (N), putative stem cells (pSC) and macrophages (M). (**B**) Higher magnification on a consecutive section of the marked area in (**A**) highlights telopodes (Tp) surrounding the blood vessel in the proximity of Schwann cells (Sch). Cardiomyocytes (CM). Bars 10 μm (**A**), 2 μm (**B**).

**Fig. 5:**
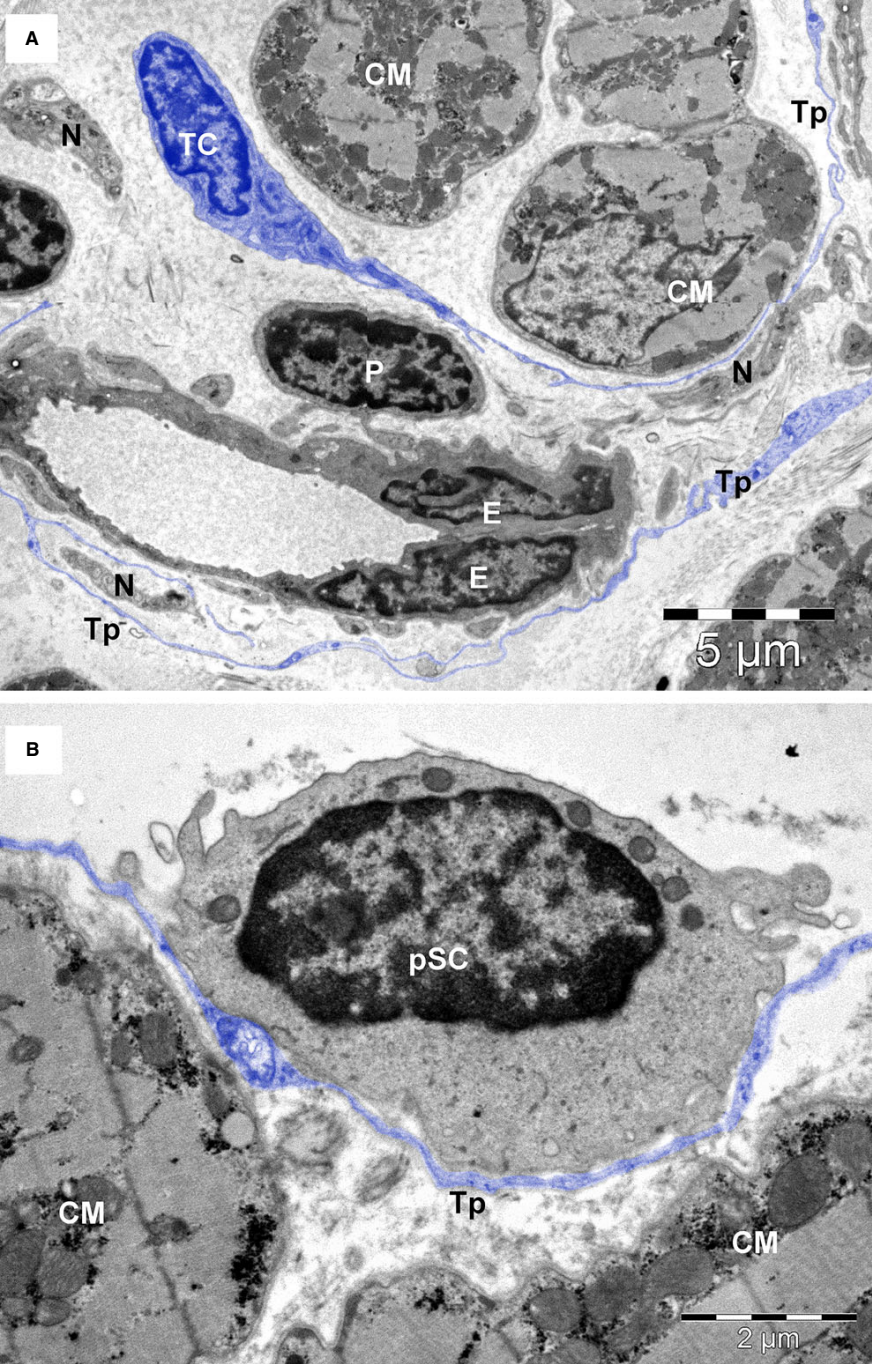
TEM images of atrial interstitium from an 8-months-old patient. (**A**) Telocytes (TC) with telopodes (Tp), capillary with endothelial cells (E) and pericyte (P), nerve endings (N). (**B**) A telopode (Tp) is enfolding a putative stem cell (pSC) with very few mitochondria and numerous ribosomes in the cytoplasm. Bars 5 μm (**A**), 2 μm (**B**).

**Fig. 6:**
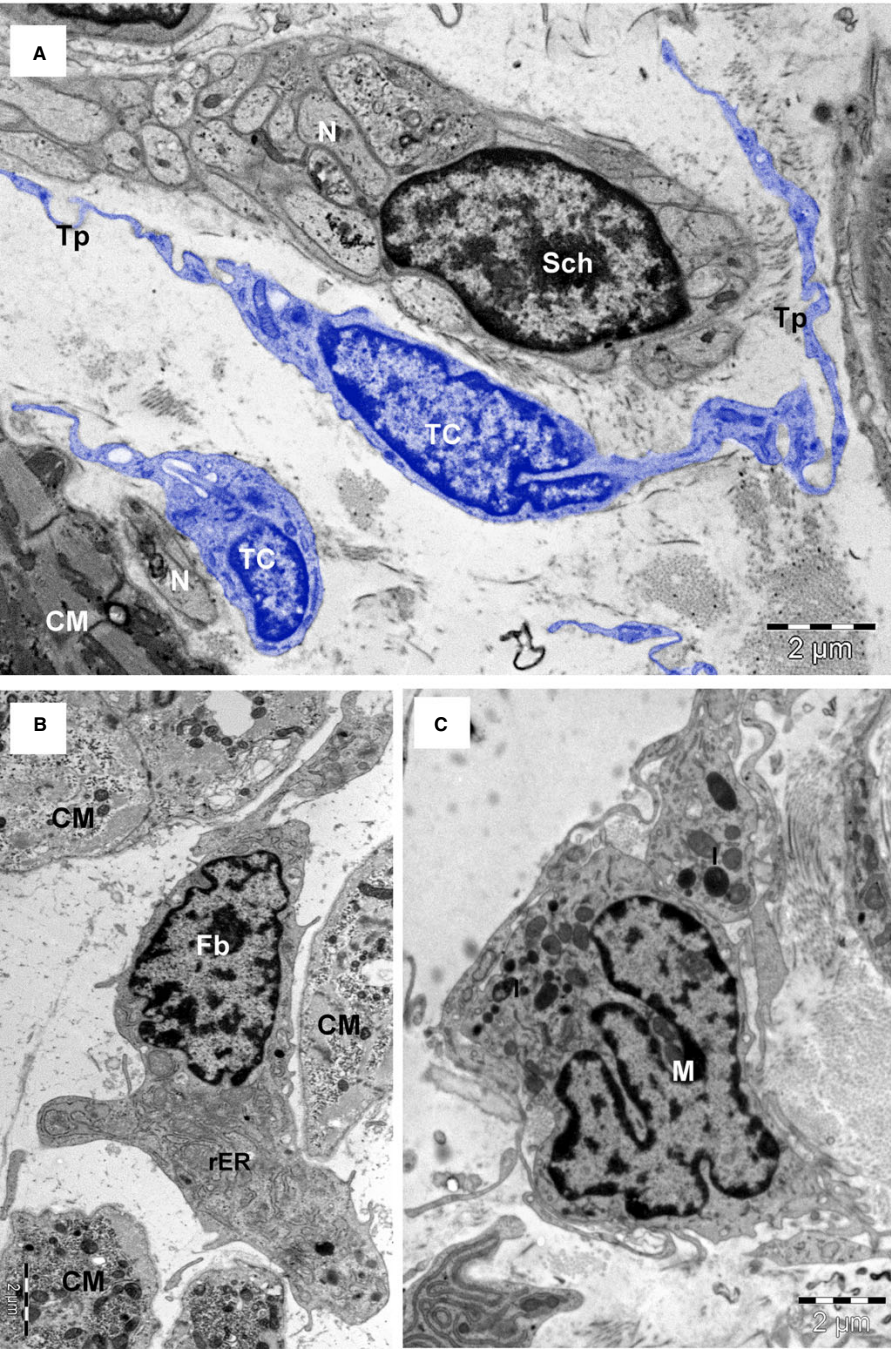
TEM images (8-months-old patient) highlight the differences between telocytes (TC) with long telopodes (Tp), and Schwann cell (Sch) (**A**); the fibroblast (Fb) with abundant rough endoplasmic reticulum (rER) (**B**) and the macrophage (M) with the cytoplasm filled with lysosomes (l), and coated pits (**C**). Bars 2 μm (**A** and **B**), 1 μm (**C**).

Table 3 shows the type of cells counted on EM micrographs and Figures 2–6 show examples of images.

Cardiomyocytes are most numerous cells in (atrial) myocardium (in terms of number/mm^2^), representing 75.7% in newborns, 88.4% in children and 85.7% in adults. Noteworthy, the differences between newborns and children or adults and between children and adults were statistically significant (*P* < 0.01 and *P* < 0.02, respectively).

Based on ultrastructural features, we identified at least eight types of interstitial cells (non-CMs) in human atrial myocardium: *endothelial cells* (Figs 2, 4, 5A), *pericytes* (Fig. 2 and 5A), *vascular smooth muscle cells* (Fig. 4), *Schwann cells with nerve endings* (Figs 4 and 6A), *fibroblasts* (Fig. 6B), *macrophages* (Fig. 6C), *TCs* (Fig. 3, 4, 5A and 6A) and *CSCs* (Fig. 2, 4A and 5B). *Mast cells* were also found in human hearts in low number, near blood vessels, and therefore mast cells were not counted. Quantitatively, mast cells represent a non-significant fraction of normal human myocardium [55]. However, the density of mast cells is markedly higher in patients with myocarditis, myocardial infarction, dilated cardiomyopathy, atherosclerosis, *etc*., when compared with humans with no cardiac pathology [56].

### Cardiomyocytes

Table 4 shows the main morphometric results when various parameters of human atrial CMs were compared as a function of age. Noteworthy, as expected, the diameter (thickness) of CMs from children and adults was similar (13–14 μm), but it was almost double in comparison with newborns. Furthermore, the mean cross-sectional area of children and adult CMs ranges from about 170 to 200 μm^2^, which is about three times larger than in newborns. Based on our data, it seems that males and females have rather similar CM volumetric data in humans.

**Table 4:** The cardiomyocyte morphometry in atria of human ageing heart (mean ± SE)

Cardiomyocyte	Newborns	Children	Adults
Diameter (μm)	7.4 ± 1.8	12.7 ± 2.1*	13.5 ± 2.3*,†
Cardiomyocyte cross-sectional area (μm^2^)	65.1 ± 14.3	166.6 ± 55*	188.3 ± 68.4*
Nucleus area (%)‡	27.1 ± 4.1	21.6 ± 5.8§	23 ± 5.3§
Euchromatin area (%)	68 ± 9.7	62 ± 9.5§	65.3 ± 8.2§
Mitochondrial area (%)	31 ± 4.9	35.1 ± 7.5*	27.3 ± 5.1§
Myofibril area (%)	47 ± 8	58 ± 8*	57 ± 12*
Sarcomere length (μm)	1.7 ± 0.1	1.4 ± 0.1§	1.4 ± 0.3§
Caveolae (no/μm)¶	1.2 ± 0.5	0.8 ± 0.2§	0.3 ± 0.02§
Lipofuscin granules (%)	–	–	3.5 ± 0.4

* The number significantly *increased* in children and adults compared with newborns (*P* < 0.01).

† The number of cells significantly *increased* in adults compared with children (*P* < 0.05).

‡ Cross-sectional area of CMs.

§ The number significantly *decreased* in children and adults compared with newborn group (*P* < 0.05).

¶ Number per μm of plasma membrane length.

#### Nucleus

After EM examination of several thousands of images of human myocardium of children and adults, we did not find clear images of mitoses. However, very rarely, we found EM aspects of mitoses in the case of a 17-day newborn. Figure 3 shows a CM undergoing *mitosis*, namely prophase. Table 4 also indicates that CM nucleus has larger cross-sectional area in newborns *versus* children and adults. Moreover, in newborns, the *euchromatic field* inside the nucleus (68 ± 9.7) is significantly larger in comparison with children (62 ± 9.5) and adults (65 ± 8.2) (in both cases *P* < 0.05).

#### Organelles

We also estimated (Table 4) the relative volumes (percentage of cell volume) of mitochondria, myofibrils, caveolae and lipofuscin granules. As expected, the amount of *mitochondria* that we found was in the range of 25–30% of cell volume, under (very) good fixation conditions: lower in newborns, higher in children and again lower in adults. Table 4 shows that *myofibrils* are the most abundant organelle, occupying 50–60% of the cell volume. Their volume is higher in children and adults (∼60%) in comparison with newborns (∼50%; *P* < 0.01). *Sarcomeres* with typical banding appeared longer in newborns (1.7 μm) when compared with children and adults respectively (1.4 μm, *P* < 0.05). Caveolae (surface microvesicles), inpocketings of sarcolemma, were counted: Table 4 indicates that ageing diminishes the number of caveolae per μm of cell membrane length.

Traditionally, it is presumed that *lipofuscin pigment granules* increase with ageing. Table 4 shows *no lipofuscin granules in CMs of newborns and children* (estimation on 600 micrographs). We found lipofuscin granules only in adults, where the pigment occupies 3.5 ± 0.4% of cell volume.

### Non-cardiomyocyte compartment (interstitial cells)

**Fig. 7:**
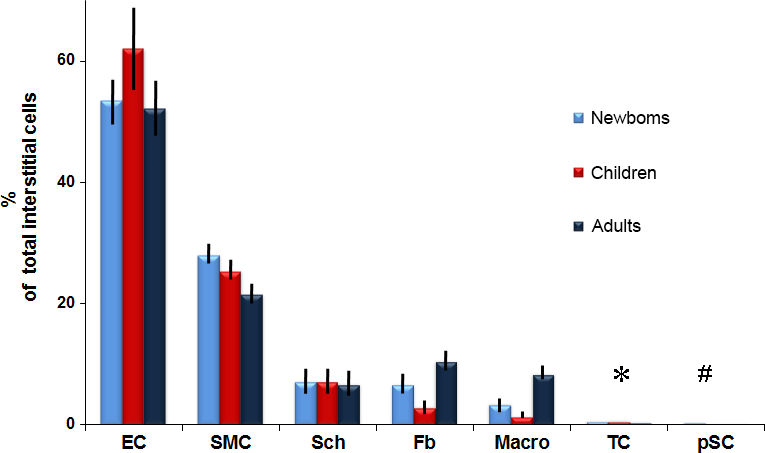
The percentage of different types of interstitial cells in the human atrial tissue and their variation on ageing processes. EC – endothelial cells; SMC – pericytes/vascular smooth muscle cells; Sch – Schwann cells; Fb – fibroblasts; Macro – macrophages; TC – telocytes; pSC – putative stem cells. The values for TCs and pSCs are as follows: * 0.6% in newborns; 0.7% in children and 0.5% in adults; # 0.5% in newborns; 0.2% in children and 0.1% in adults.

Figure 7 shows percentage of the main cellular components that occupy interstitium between CMs: endothelial cells (52.2–62.1%), vascular smooth muscle cells and pericytes (21.6–28.1%), Schwann cells with nerve endings (6.7–7.2%), fibroblasts (3–10.5%), macrophages (1.4–8.4%), TCs (0.5–0.7%) and putative stem cells (0.1–0.5%). The majority of non-CMs are vascular cells.

Endothelial cells are easily recognized by EM (Figs 2, 4 and 5A). These cells contain few organelles and a large variety of vesicles, caveolae and coated pits involved in endocytosis and transcytosis [57]. The abluminal face of endothelial cells is covered by a basal lamina.

Vascular smooth muscle cells (Fig. 4) surround the endothelial cells. The presence of thin actin filaments with dense bodies and numerous caveolae alternating with dense plaques establish electron microscopic diagnosis [58].

Pericytes (Figs 2 and 5A) represent a type a mural cell, principally located in microvasculature. They occasionally encircle the blood capillaries, but may be found at the level of pre-capillary arterioles and post-capillary venules [59,60]. Pericytes (Fig. 3) showed a large heterochromatic nucleus, small Golgi complex, mitochondria, rough endoplasmic reticulum as well as microtubules and filaments extending in their processes. Pericytes are embedded in the basal lamina of endothelial cells. For practical reasons, pericytes were counted together with vascular smooth muscle cells. Interestingly, Nees [61] suggested that pericyte together with endothelial cells and TCs represent a ‘functional unit’.

Schwann cells (Figs 4 and 6A) surrounding nerve endings were often found in the cardiac interstitium and presented a continuous basal lamina. Their cytoplasm contains few microtubules and no contractile filaments. Schwann cells represent the third numerous population of interstitial cells and their number remained constant in all three groups: newborns, children or adults (Table 3). Nonetheless, non-myelinated nerve processes are plentiful in the myocardium [2].

Fibroblasts (Fig. 6) are very easily and precisely identified by EM. Fibroblasts have a large and pleomorphic cellular body with short processes, an oval and slightly euchromatic nucleus (with 1–2 visible nucleoli), abundant rough endoplasmic reticulum and prominent Golgi complex. Fibroblasts have no external lamina and are usually associated with collagen fibrils. Figure 7 shows that fibroblasts, in adult human myocardium, are only about 10% of non-CM interstitial cells, a percentage comparable with Schwann cells or macrophages.

We cannot confirm that cardiac fibroblasts are the most prevalent cell type in the heart as Porter and Turner [62] assumed, or that cardiac fibroblasts are arranged in sheets and strands that run in parallel with CMs [63].

Myofibroblasts, a ‘fibroblast–smooth muscle cell hybrid’, do not exist or are extremely difficult to find in healthy human heart [64]. However, within days after an injury, myofibroblasts appear in abundance [49,65]. We did not find myofibroblasts in our specimens although the electron microscopic criteria to recognize these cells are quite simple.

Macrophages (Fig. 5) were identified based on their numerous endocytotic vesicles and lysosomes. Macrophages represent below 10% of interstitial cells in human adult myocardium (Fig. 7). Sometimes, in ultrastructural studies, the cardiac macrophages are overlooked [*e.g*. ref. [2]] although they are protagonist immune cells and other experimental methods showed that cardiac macrophages increase during disease [66,67].

Telocytes are shown in Figures 2, 4, 5 and 6A and their ultrastructural morphometry in Table 5. TCs were recognized based on the short definition: ‘cells with Tps’ [16,21,48]. These Tps are very long processes (several tens of micrometres), which are convoluted and have a dichotomic branching pattern. Tps show (on ultrathin sections) an alternance of thin segments called podomers and dilated segments named podoms (Table 5). The latest contain mitochondria and elements of endoplasmic reticulum. TCs have a small cell body (Table 5) containing an oval/round nucleus, which is slightly heterochromatic. The rim of cytoplasm surrounding the nucleus accommodates mitochondria (their number depending on age), endoplasmic reticulum cisternae and a small Golgi complex.

**Table 5:** Morphometry of cardiac telocytes

	Newborns	Children	Adults
Cell body diameter (μm)	5.7 ± 2.6	6.5 ± 3*	5.7 ± 2.3
Nucleus (euchromatin area %)	49 ± 9.3	52 ± 8.5*	48 ± 7.9
Mitochondrial area (%)	3.6 ± 0.5	2.8 ± 0.9†	2 ± 0.8†
Telopode (thickness μm)			
Podomers	0.1 ± 0.04	0.1 ± 0.05	0.1 ± 0.06
Podoms	0.7 ± 0.3	0.90 ± 0.4	0.8 ± 0.3
Caveolae (number/μm)	0.82 ± 0.25	1.02 ± 0.46	1.30 ± 0.62‡
Extracellular vesicles number§	3 ± 1	5 ± 2*	2 ± 1

* The number significantly *increased* in children compared with newborns (*P* < 0.02).

† The number significantly *decreased* in children and adults compared with newborn group (*P* < 0.01).

‡ The number significantly *increased* in adults compared with newborns (*P* < 0.01).

§ The number of extracellular vesicles found around the telocyte within a distance of 0.5 μm from cell membrane.

Numerically, we found that TCs represent a small fraction of human cardiac interstitial cells in the range of 0.5–1%. However, two things should be considered. Firstly, TCs are more numerous than CSCs and secondly, because of their extensive Tps, TCs are occupying a volume fraction much larger than their cell bodies.

Putative stem cells (Figs 2, 4A and 5B) were considered all cells without any distinctive features, with a large nucleus, small number of mitochondria, few endoplasmic reticulum cisternae and numerous free ribosomes (Fig. 5B). The CSCs have no prolongations. The stem cells that we are describing here share similar properties with cardiac cells derived from human embryonic and induced pluripotent stem cells [68]. We found that the putative stem cells represent only 0.01% and 0.1% from total atrial CMs in adults and newborns, respectively (Table 3). If we consider only interstitial cells (Fig. 7), the number of stem cells is decreased fivefold by age (from 0.5% to 0.1%) in newborns *versus* adults, respectively.

## Discussion

Our results (Figs 2–6) show that the human heart fragments (taken from atrial appendages) were properly fixed for transmission electron microscopy. No ‘empty’ spaces were found inside CMs. Normal CMs, as well as interstitial cells (Table 3), were easily identified. Table 4 shows that the characteristic morphological features of normal human CMs were preserved: (*i*) the mean cross-sectional area of CMs is about 180 μm^2^; (*ii*) myofibrils are the most abundant organelles, occupying approximately 50–60% of cytoplasm; (*iii*) sarcomere length is about 1.5 μm; (*iv*) mitochondria are aligned in rows between myofibrils and occupy 25–35% of CM cytoplasm; and (*v*) the nucleus is prominent with the predominance of active, euchromatin [2,69–71].

### Is the human myocardium a post-mitotic pump?

From the point of view of organ biology, the cardiac muscle is considered a post-mitotic entity. It has been difficult to establish whether we are limited to the CMs we are born with or if CMs are generated also later in life [72]. Counting of CMs in different species is problematic and, moreover, the traditional methods used to study CMs proliferation have a number of limitations [73]. Although contradictory results were reported [72,74–83], now the dominant idea is that the majority of CMs are terminally differentiated because of irreversible cell cycle arrest [84]. In humans, post-natal CM growth is primarily hyperplastic for the first months after birth [85] (see also Table 3 and Fig. 3 showing mitosis in myocardium of a 17-day newborn). After several weeks/months, CMs exit the cell cycle and terminally differentiate by hypertrophic growth, which may coincide with CMs binucleation. Indeed, as Gerdes [2] also reported, we have never observed single example of a dividing myocyte nucleus in human adult heart, despite extensive screening of whole tissue.

### Cell modifications during ageing

Light microscopy (Fig. 1) showed that interstitial area (*non-myocytic space*) increased progressively with age, presumably because of accumulation of extracellular matrix and proliferation of interstitial cells, primarily fibroblasts and macrophages. The interstitial compartment increased in adults *versus* newborns with about 10%. It is also to be mentioned that the number of *blood capillary* increased (per mm^2^) (Table 2) several hundreds in children and adults *versus* newborns, although the capillary lumen did not increase.

### Cardiomyocytes

An important finding of our results is that CMs are the most numerous cells in myocardium (in terms of number/mm^2^) representing 76%, 88% and 86% in newborns, children and adults respectively. This is in contradiction with the fact that non-myocytes were supposed to make up over 75% of total number of cells in heart [2].

Table 4 shows for comparison, the main morphometric parameters of CMs as a function of age. The *diameter* (thickness) of human adult CMs (14–15 μm) is in full agreement with the accepted data [86]. Noteworthy, the diameter of CMs in newborns was only one half (7.5 μm). The smaller radius might reflect more intense metabolic exchanges. Moreover, the radius increase in children may be the result of the transition from hyperplasia (the first few weeks/months) to hypertrophy (early childhood).

The area occupied by *mitochondria* in CMs is maximum in children and decreases gradually during the adult life. The high content of mitochondria (presumably the highest among the human cells) explains the fact that CM is the most physically energetic cell in body, contracting constantly, without tiring, 3 billion times or more in an average human lifespan [87].

The area occupied by *myofibrils* remains constant in children and adults, but significantly higher (*P* < 0.01) than in newborns. The number of *caveolae* is significantly lower in adults *versus* newborns or children suggesting modifications of Ca^2+^ movements as caveolae together with sarcoplasmic reticulum and mitochondria represent Ca^2+^-releasing/accumulating units [58].

Last but not least, we did not find *lipofuscin pigment granules* in CMs of newborns and children (Table 4). The absence of lipofuscin (‘wear and tear pigment’) in newborns and children indicates that ageing starts during adulthood.

### Interstitial cells (non-CMs)

Table 3 shows that numerically non-CMs represent less than 10% of the total cell population. Most of these non-CMs are vascular cells (endothelial cells, pericytes and vascular smooth muscle cells).

*Schwann cells* (Fig. 7) surrounding unmyelinated nerve endings represent about 7% of total interstitial (non-myocytic) cells, which is an unexpected fact. Usually, the Schwann cells have been overlooked, at least in ultrastructural studies. It is noteworthy that, the number of Schwann cells remains constant during life (Fig. 7).

In recent years, the dynamic and complex interactions of *fibroblasts* with CMs have become a focus of investigation [88]. However, our findings (Table 3) do not support the concept that fibroblasts are the most numerous cell type in the normal adult heart [89,90] and account for about 20% of the myocardial volume [7]. Also, we could not find evidence for the supposition [91] that the normal adult heart contains 70% non-CMs, of which the majority is cardiac fibroblasts. If we think that fibroblasts are normal cells, producing collagen fibrils and some components of extracellular matrix, then the results mentioned above appear questionable.

Figure 7 shows that the number of fibroblasts ( of intercellular non-myocytic cells) is less than 5%. However, in adults, fibroblasts may represent about 10% of intercellular cells. This fact could be ascribed to various injuries to adult heart that result in a degree of fibrosis. Myofibroblasts could be the apex of the fibrotic phenotype and the principal source of the extensive extracellular matrix [92]. Undoubtedly, the ageing enlarges the intercellular spaces (Fig. 1).

Figure 7 shows that the number of *macrophages* is at least two times higher in newborns in comparison with children. This result could be expected as cardiac macrophages presumably provide the necessary signals to drive angiogenesis and regeneration of the neonatal mouse heart [93]. The significant increase in macrophage number in adult myocardium (about 8% of interstitial cells) might be ascribed to various antigenic aggressions during adulthood. However, the conventional EM cannot discriminate between the diverse functional subpopulations of resident macrophages in the adult heart. For instance, Epelman *et al*. [94] identified four subpopulations of cardiac macrophages, based on different cell surface markers.

Although the number of *TCs* decreases significantly in adults *versus* children (Fig. 7), no considerable ultrastructural changes were found (Table 5). For example, the total area of mitochondria is decreased and the number of caveolae is higher. The thickness of podomers and podoms (on sections) remains unchanged. The 3D imaging by FIB-SEM tomography should be used to reveal the actual morphology of Tps during ageing.

In addition, dynamics of cardiac Tps can be influenced *in vitro* by the proteic composition of the extracellular matrix [29]: the stronger spreading being produced by fibronectin, while the lowest by laminin. Collagen determined the highest dynamics of Tps extensions. Low-level laser stimulation increased telopodal lateral extensions in cell culture [95] and some implications may emerge for low-level laser therapy.

From the morphopathology point of view, TCs appear implicated in at least two diseases. In the isolated atrial amyloidosis, which appears in patients with long-standing atrial fibrillation, Tps surround the amyloid deposits limiting their spreading into the interstitium [96]. In addition, TCs were almost completely absent in fibrotic areas of systemic sclerosis myocardium [97].

### Human cardiac stem cells

Nevertheless, we are still at the beginning of a therapeutic revolution for myocardial infarction or heart failure. Many important issues of action of CSCs, long-term engraftment, optimal cell type(s), and dose, route, and frequency of cell administration remain to be resolved and no cell therapy has been conclusively shown to be effective [98–105].

The ‘tandem’ TCs-CSCs apparently challenges the ‘dogma’ that cardiac regeneration/repair of myocardial infarction or heart failure might be performed by CSCs *only*. Strong arguments against the dogma are provided by comparative biology: the high number of TCs has been found in association with CMs in zebrafish or newt heart [13]. In these species, after amputation of the ventricle apex, the heart regenerates and TCs are the first cells involved in this process [106].

Significant for our subject is that using EM and electron tomography we have found nanoscopic junctions between TCs and CMCs, as well as between TCs and CM progenitors in CSC niches [18]. The intermembrane distance about 10–20 nm, is in the range of macromolecular interactions. In addition, we can mention that in stem-cell niches (in mammals and humans), TCs *always* do exist in close contacts with stem cells, for example: lungs [107], skeletal muscle [108], skin dermis [109], meninges and choroid plexus [110], limbus and uvea of eye [111] or liver [112,113].

In conclusion, our studies (*e.g*. Fig 5B) underline the fact that TCs and CSCs form a structural and functional unit, a ‘tandem’ [11,18,48]. This suggests that preconditioning of CSCs with TCs could be useful before or during cell transplantation.

Last but not least, the number of CSCs (interstitial space) decreases fivefold from newborns to adults (Fig. 7). This may explain the limited regenerative and/or reparative ability of “adult myocardium”. It seems attractive to use TCs in ways expected to contribute in development of age-intervention protocols [114], especially because proteomics [40] showed a high expression of super oxide dismutase (SODM) in TCs.
